# Supplementary material for: Mechanism of Phase Separation in Aqueous Two-Phase Systems
Source: Int J Mol Sci. 2022 Nov 19;23(22):14366. doi: 10.3390/ijms232214366 (PMC9697872; doi:10.3390/ijms232214366)
Supplement: Supplementary file 1 [file ijms-23-14366-s001.zip › ijms-1982817-supplementary.pdf]

Supplementary Data for Mechanism of phase separation in aqueous two-phase systems

Amber R Titus, Pedro P Madeira, Luisa A Ferreira, Vladimir Y Chernyak, Vladimir N Uversky, Boris Y Zaslavsky

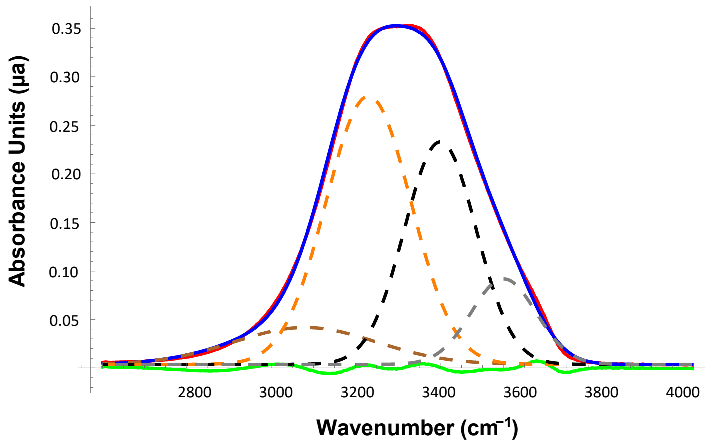

Figure S1. Example of the ATR-FTIR spectra of OH-stretch band in the solvent for 0.15 M NaCl in 0.01 M Na-phosphate buffer, pH 7.4 (PBS) adapted from [1]. The blue line is the measured absorption spectrum, and the red envelope is the best fit of the sum of four Gaussian components (dashed lines at positions (3080 cm^{-1} (dark orange), 3230 cm^{-1} (orange), 3400 cm^{-1} (black), and 3550 cm^{-1} (gray)). Experimental data and fit are visually almost indistinguishable.

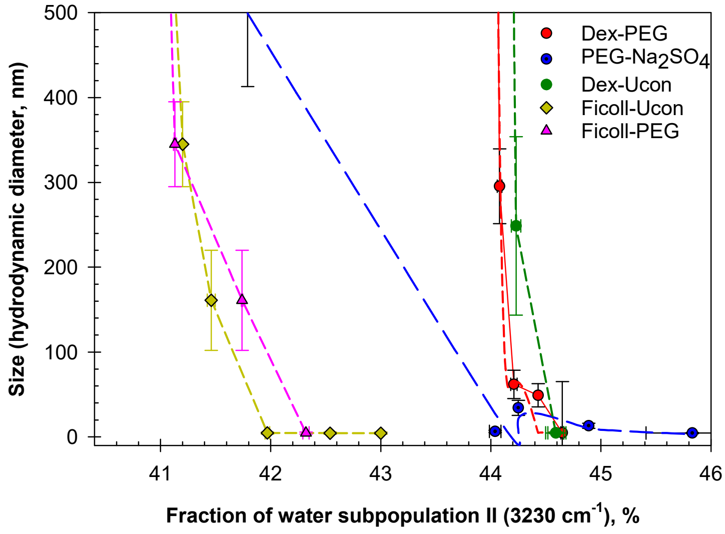

Figure S2. Size of agglomerates <500 nm formed in the mixtures of Dextran-75/PEG-8000 (red), PEG-8000/Na2SO4 (blue), Dextran-75/Ucon-3930 (dark green), Ficoll-70/Ucon-3930 (yellow), and Ficoll-70/PEG-8000 (pink) as a function of the fraction of the water subpopulation II in the mixtures. Data taken from Figure 9 in the main text.

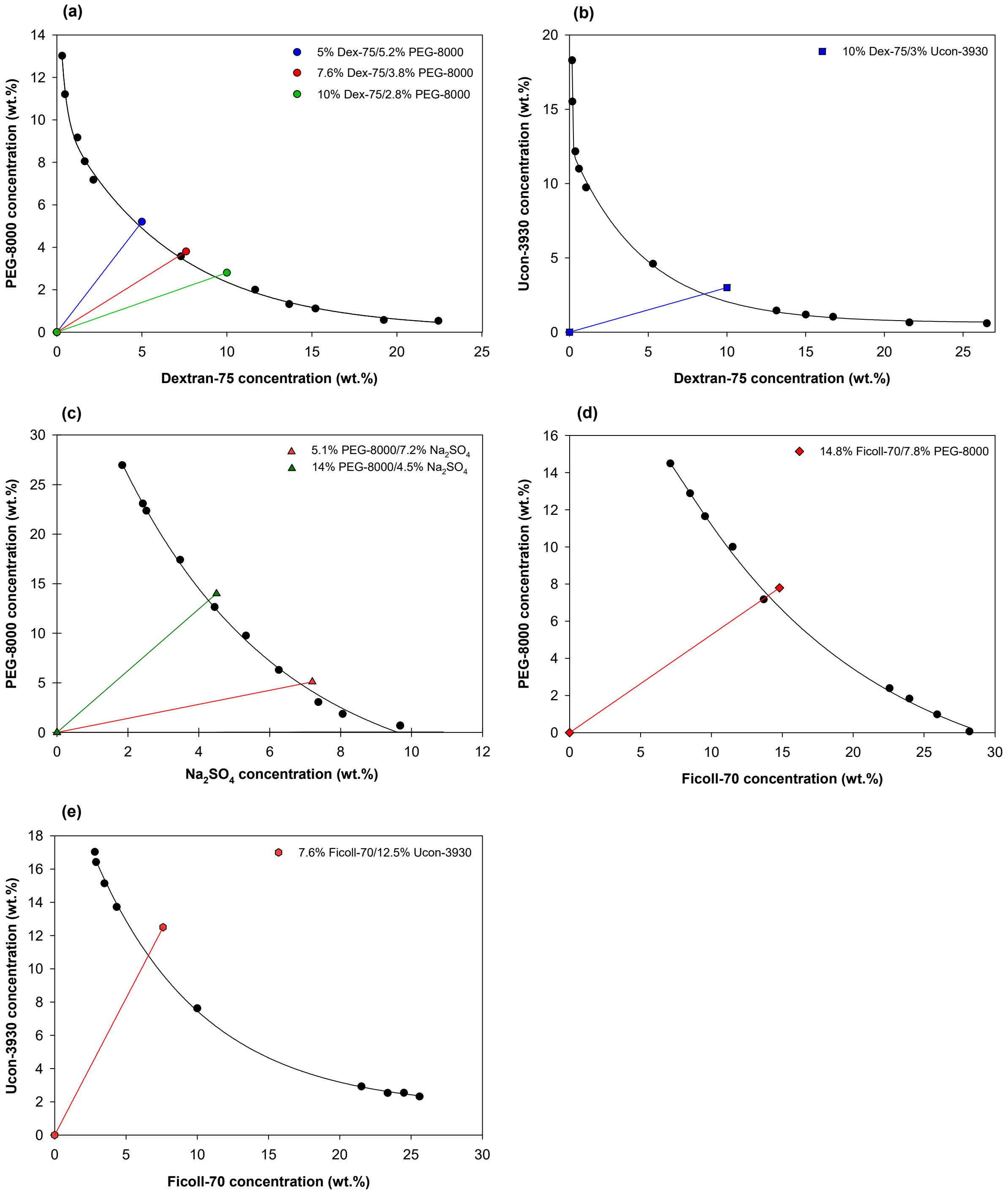

Figure S3. Phase diagrams of (a) Dextran-75/PEG-8000, (b) Dextran-75/Ucon-3930, (c) PEG-8000/Na_2SO_4, (d) Ficoll-70/PEG-8000, and (e) Ficoll-70/Ucon-3930 adapted from [2].

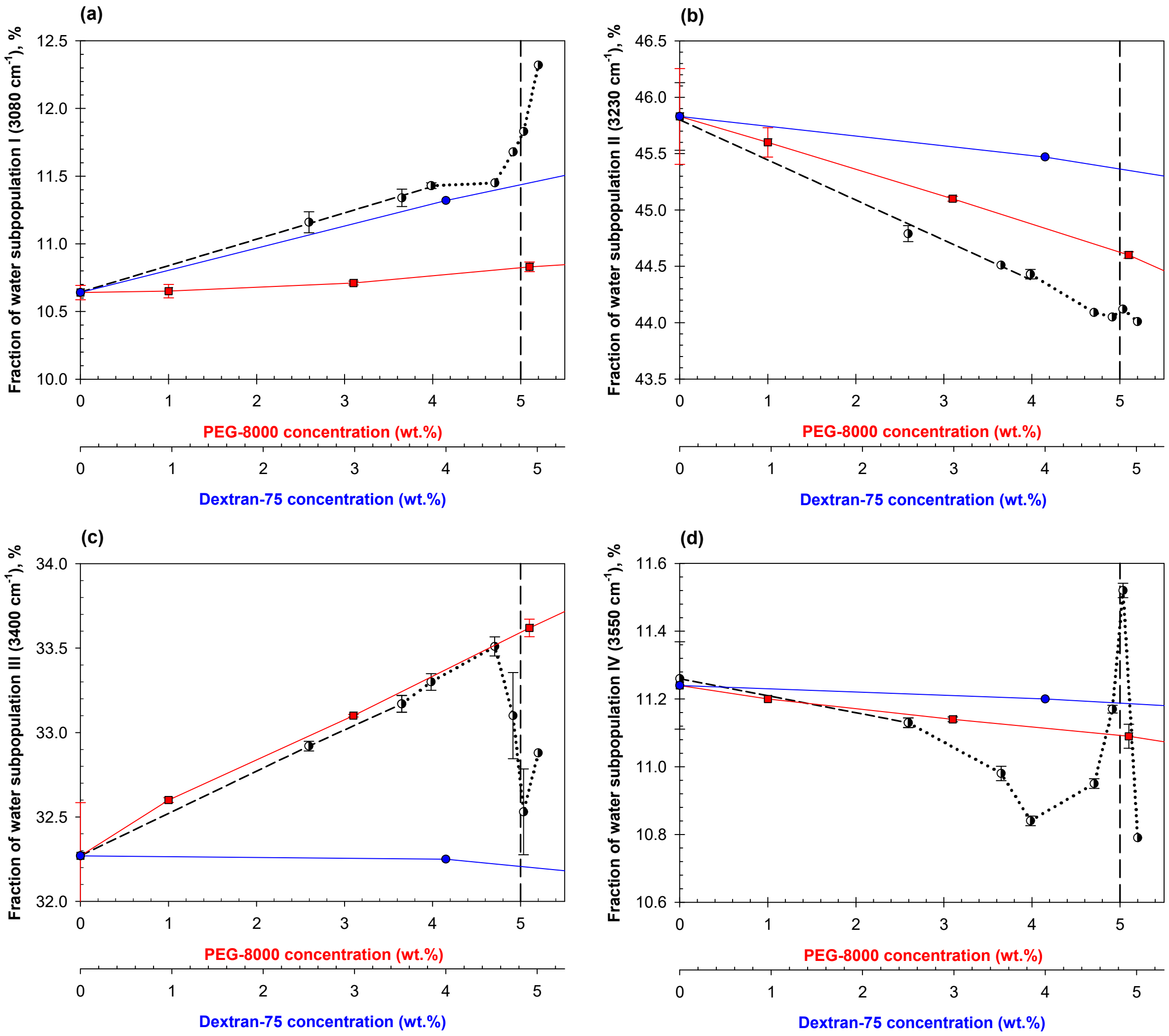

Figure S4. Concentration dependences of the water subpopulations fractions represented by the relative contribution of Gaussian components I (a), II (b), III (c), IV (d) on dilutions of mixed ATPS in PBS consisting of 5% Dextran / 5.2% PEG (black), PEG-8000 in PBS (red squares), and Dextran-75 in PBS (blue circles). The vertical dashed line represents the estimated disruption point of this system according to its phase diagram.

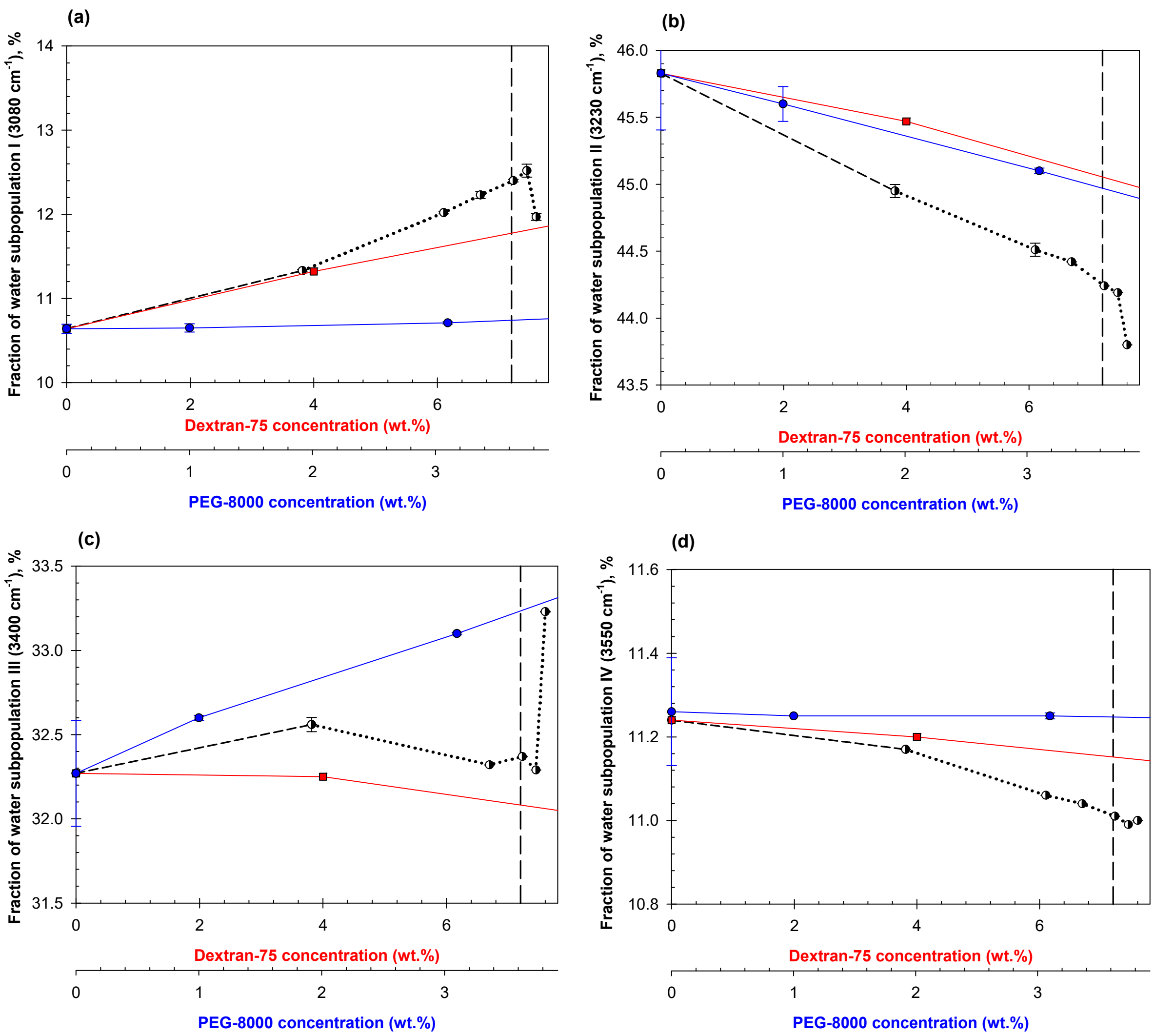

Figure S5. Concentration dependences of the water subpopulations fractions represented by the relative contribution of Gaussian components I (a), II (b), III (c), IV (d) on dilutions of mixed ATPS in PBS consisting of 7.6% Dextran / 3.8% PEG (black), Dextran-75 in PBS (red squares), and PEG-8000 in PBS (blue circles). The vertical dashed line represents the estimated disruption point of this system according to its phase diagram.

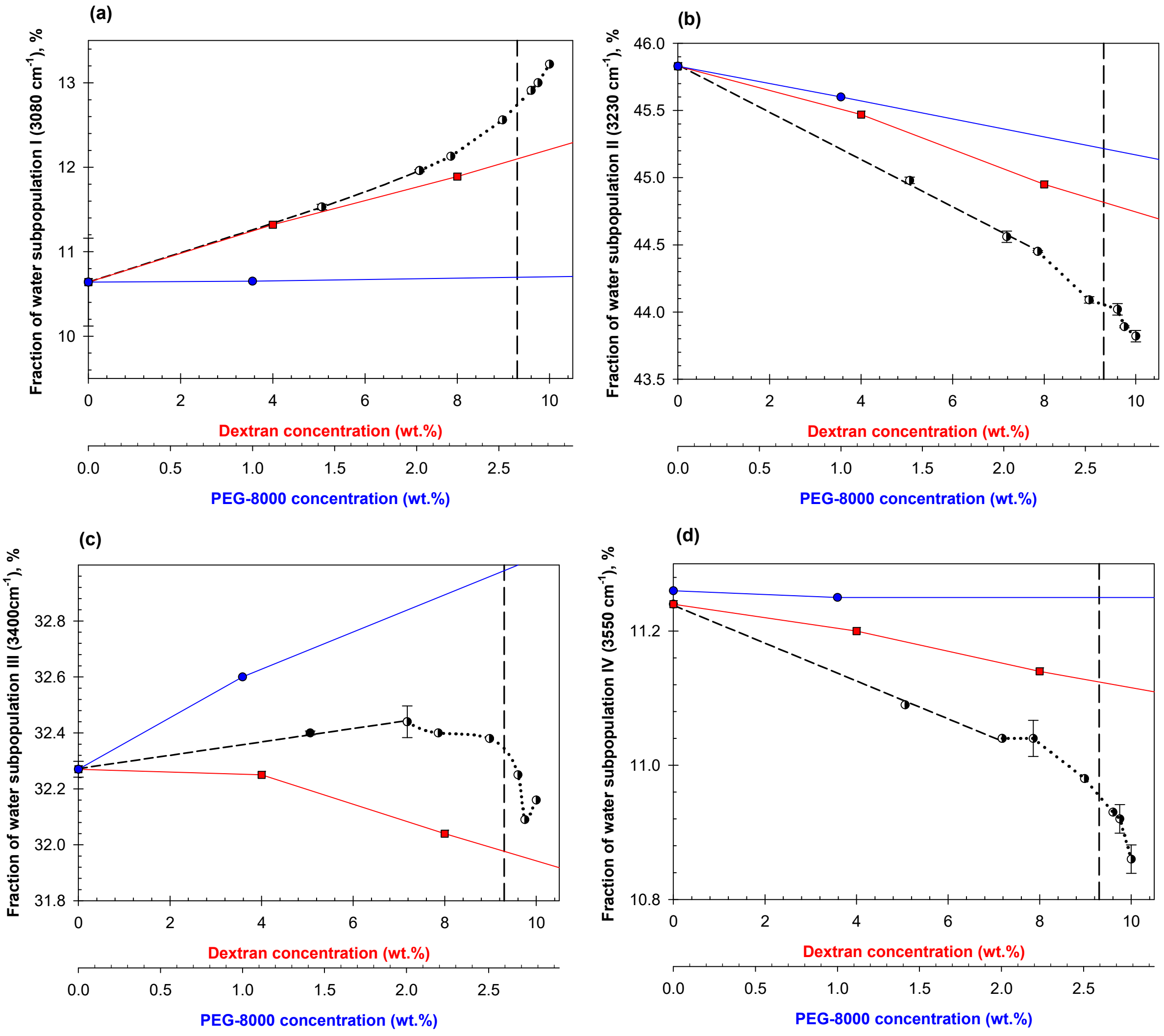

Figure S6. Concentration dependences of the water subpopulations fractions represented by the relative contribution of Gaussian components I (a), II (b), III (c), IV (d) on dilutions of mixed ATPS in PBS consisting of 10% Dextran / 2.8% PEG (black), Dextran-75 in PBS (red squares), and PEG-8000 in PBS (blue circles). The vertical dashed line represents the estimated disruption point of this system according to its phase diagram.

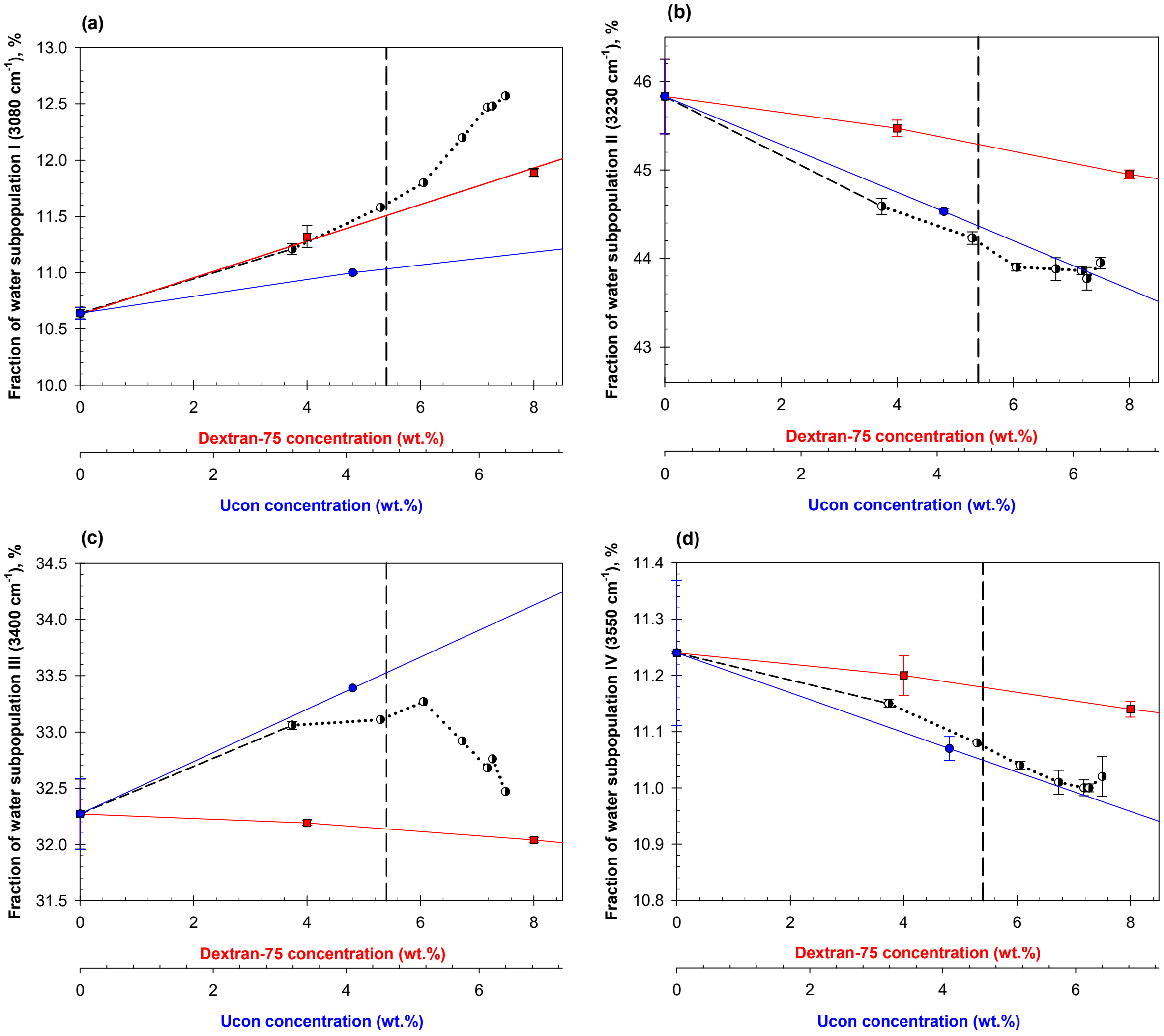

Figure S7. Concentration dependences of the water subpopulations fractions represented by the relative contribution of Gaussian components I (a), II (b), III (c), IV (d) on dilutions of mixed ATPS in PBS consisting of 7.5% Dextran / 6.2% Ucon (black), Dextran-75 in PBS (red squares), and Ucon-3930 in PBS (blue circles). The vertical dashed line represents the estimated disruption point of this system according to its phase diagram.

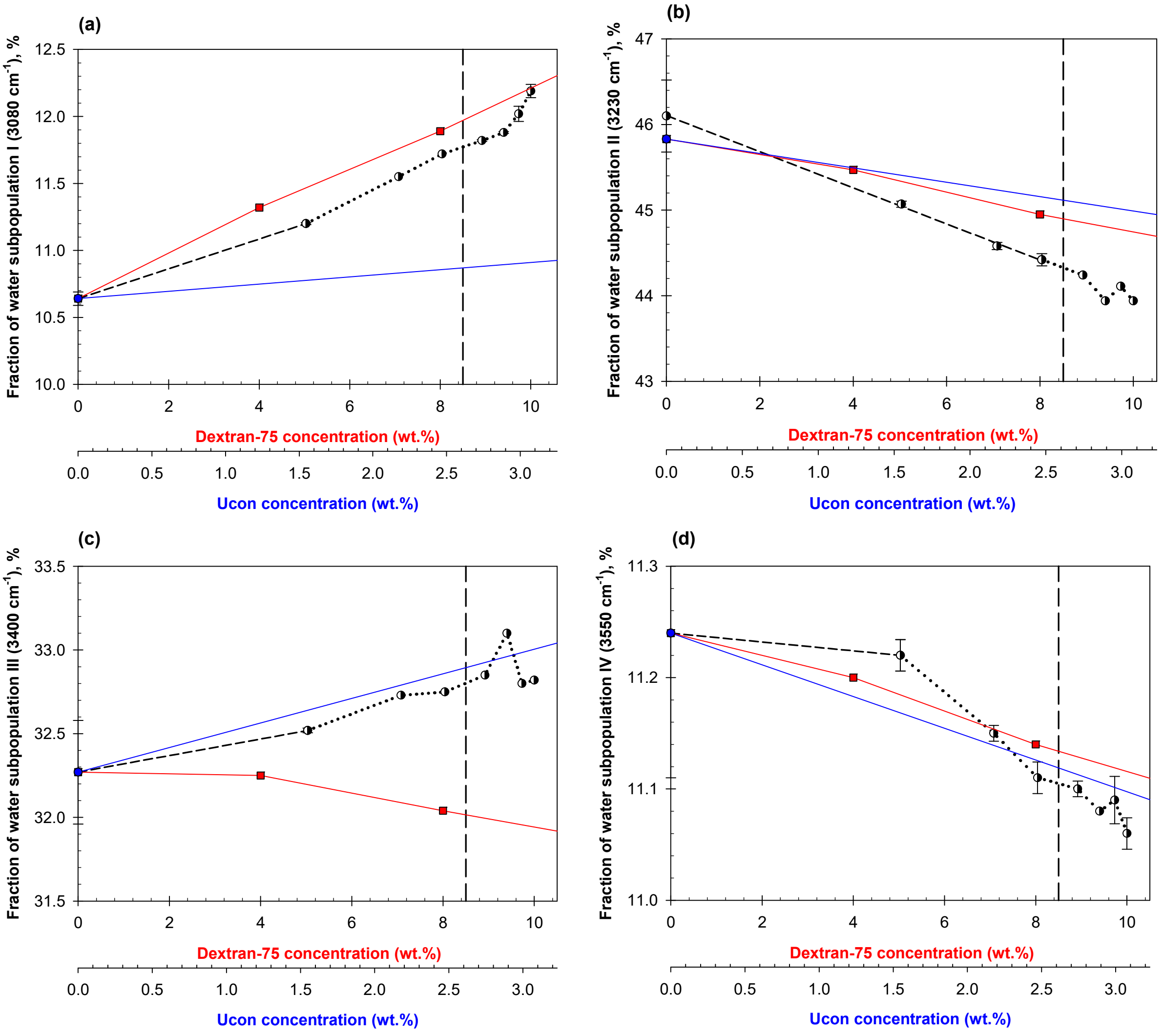

Figure S8. Concentration dependences of the water subpopulations fractions represented by the relative contribution of Gaussian components I (a), II (b), III (c), IV (d) on dilutions of mixed ATPS in PBS consisting of 10% Dextran / 3% Ucon (black), Dextran-75 in PBS (red squares), and Ucon-3930 in PBS (blue circles). The vertical dashed line represents the estimated disruption point of this system according to its phase diagram.

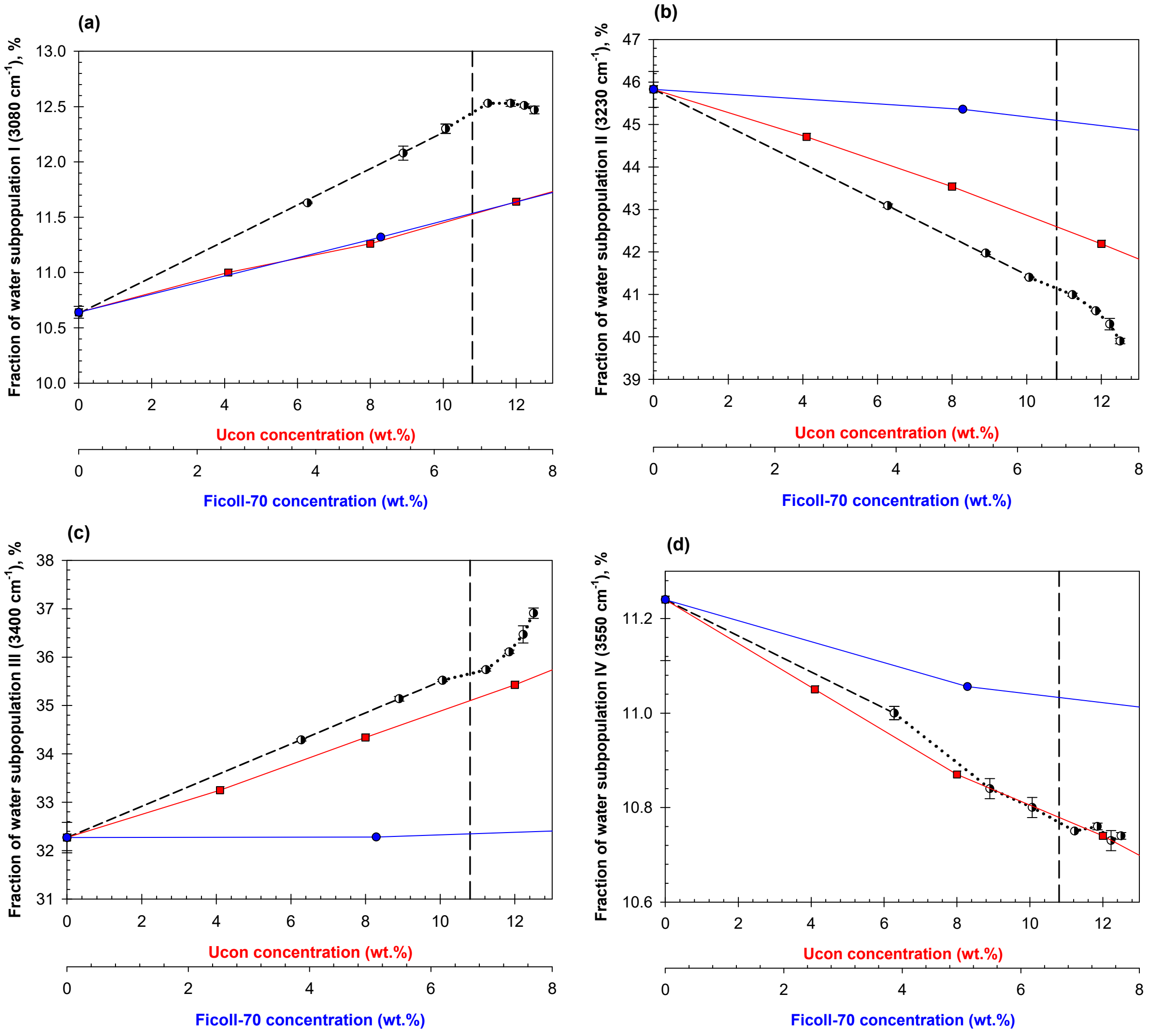

Figure S9. Concentration dependences of the water subpopulations fractions represented by the relative contribution of Gaussian components I (a), II (b), III (c), IV (d) on dilutions of mixed ATPS in PBS consisting of 7.6% Ficoll / 12.5% Ucon (black), Ucon-3930 in PBS (red squares), and Ficoll-70 in PBS (blue circles). The vertical dashed line represents the estimated disruption point of this system according to its phase diagram.

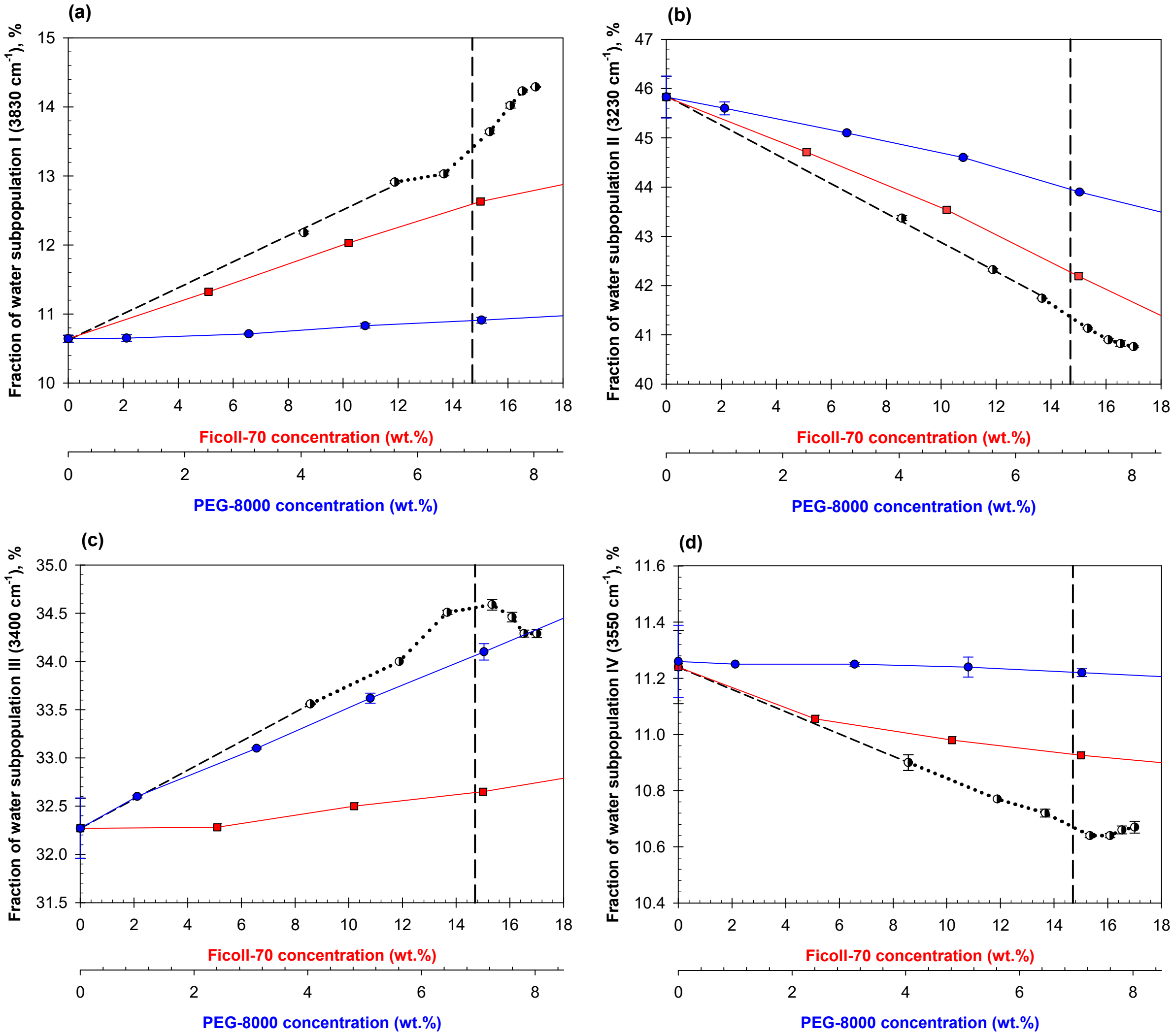

Figure S10. Concentration dependences of the water subpopulations fractions represented by the relative contribution of Gaussian components I (a), II (b), III (c), IV (d) on dilutions of mixed ATPS in PBS consisting of 17% Ficoll / 8% PEG (black), Ficoll-70 in PBS (red squares), and PEG-8000 in PBS (blue circles). The vertical dashed line represents the estimated disruption point of this system according to its phase diagram.

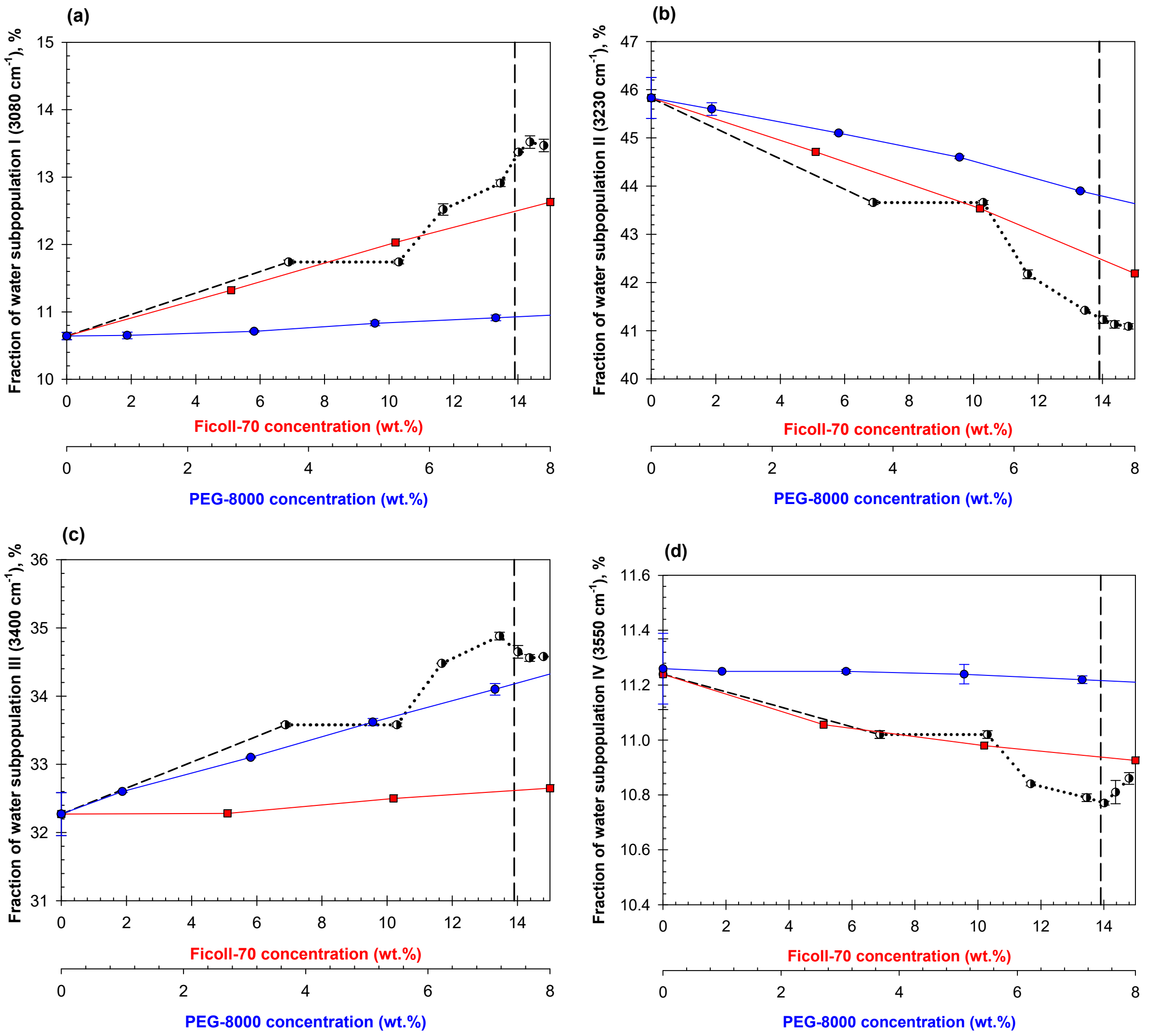

Figure S11. Concentration dependences of the water subpopulations fractions represented by the relative contribution of Gaussian components I (a), II (b), III (c), IV (d) on dilutions of mixed ATPS in PBS consisting of 14.8% Ficoll / 7.8% PEG (black), Ficoll-70 in PBS (red squares), and PEG-8000 in PBS (blue circles). The vertical dashed line represents the estimated disruption point of this system according to its phase diagram.

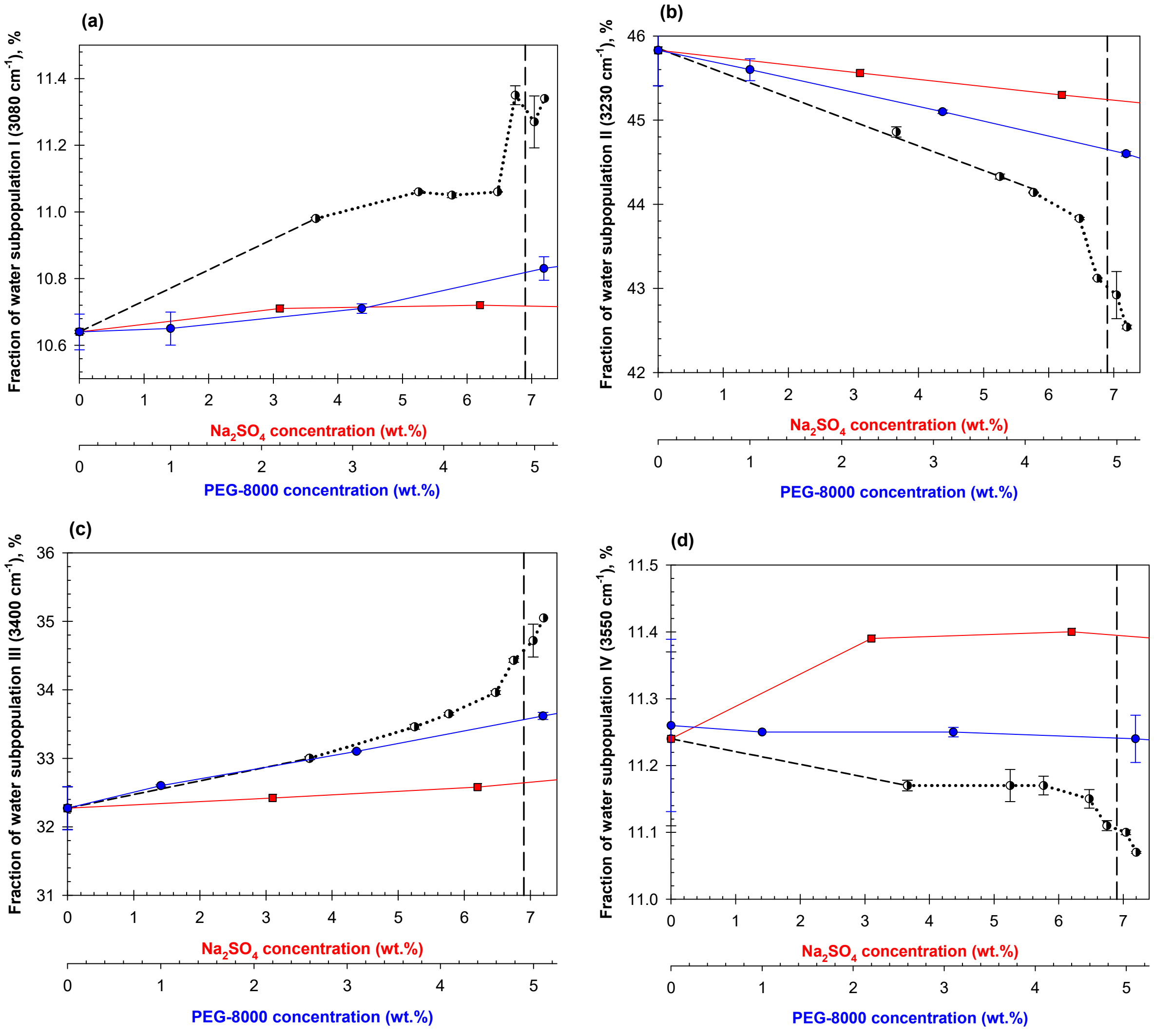

Figure S12. Concentration dependences of the water subpopulations fractions represented by the relative contribution of Gaussian components I (a), II (b), III (c), IV (d) on dilutions of mixed ATPS in PBS consisting of 7.2% Na_2SO_4 / 5.1% PEG (black), Na_2SO_4 in PBS (red squares), and PEG-8000 in PBS (blue circles). The vertical dashed line represents the estimated disruption point of this system according to its phase diagram.

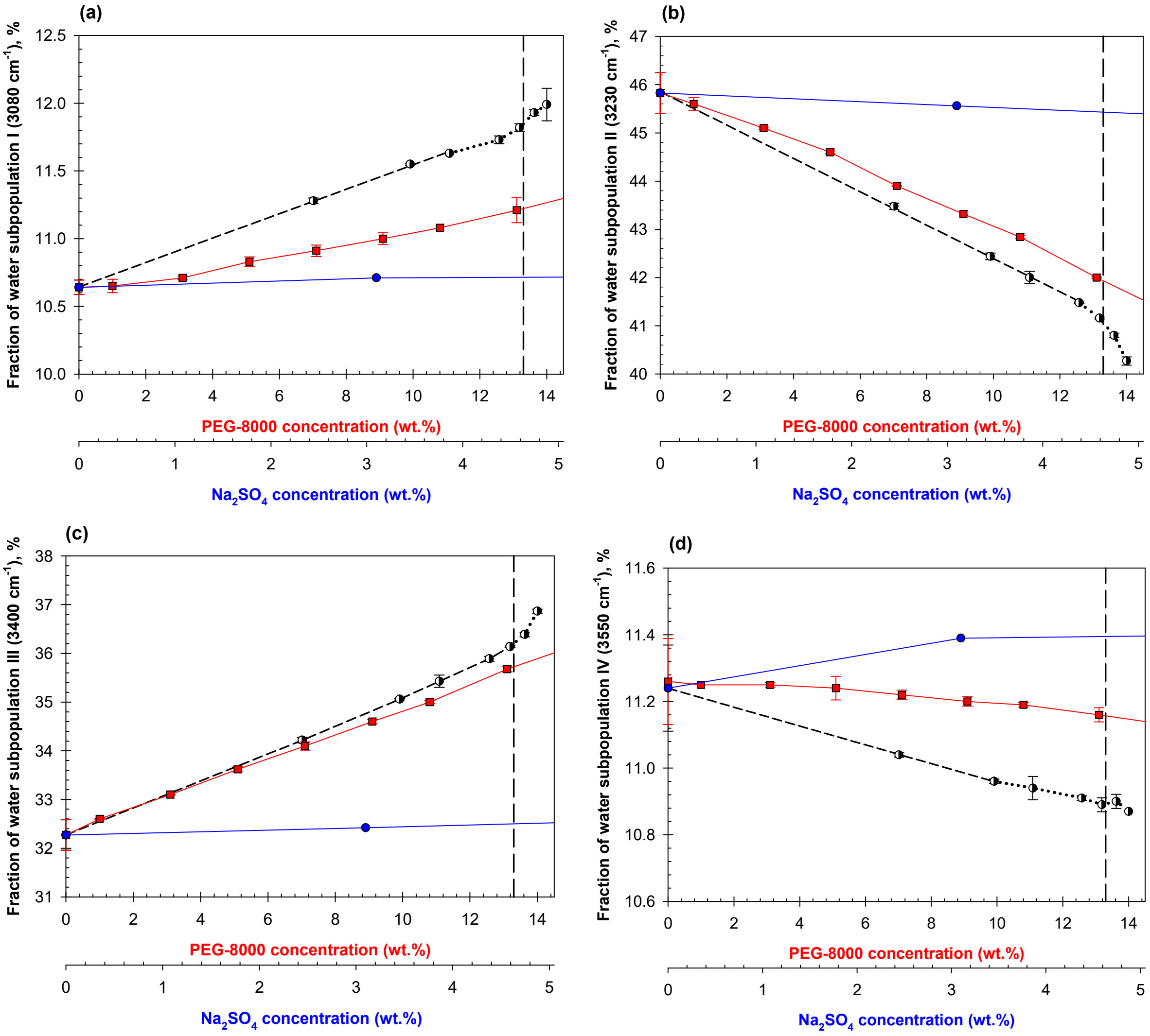

Figure S13. Concentration dependences of the water subpopulations fractions represented by the relative contribution of Gaussian components I (a), II (b), III (c), IV (d) on dilutions of mixed ATPS in PBS consisting of 4.5% Na_2SO_4 / 14% PEG (black), PEG-8000 in PBS (red squares), and Na_2SO_4 in PBS (blue circles). The vertical dashed line represents the estimated disruption point of this system according to its phase diagram.

References

[1] Titus, A.R., Madeira, P. P.; Ferreira, L. A.; Belgovskiy, A. I.; Mann, E. K.; Mann Jr., J. A.; Meyer, W. V.; Smart, A. E.; Uversky, V. N.; Zaslavsky, B. Y., Arrangement of hydrogen bonds in aqueous solutions of different globular proteins. Int. J. Mol. Sci. 2022, 23, p. 11381.

[2] Madeira, P.P., et al., Correlations between distribution coefficients of various biomolecules in different polymer/polymer aqueous two-phase systems. Fluid Phase Equilibria, 2008. 267(2): p. 150-157.
